# Association of Retinal and Macular Damage with Brain Atrophy in Multiple Sclerosis

**DOI:** 10.1371/journal.pone.0018132

**Published:** 2011-04-08

**Authors:** Jan Dörr, Klaus D. Wernecke, Markus Bock, Gunnar Gaede, Jens T. Wuerfel, Caspar F. Pfueller, Judith Bellmann-Strobl, Alina Freing, Alexander U. Brandt, Paul Friedemann

**Affiliations:** 1 NeuroCure Clinical Research Center, Charité - Universitaetsmedizin Berlin, Berlin, Germany; 2 Sophisticated Statistical Analysis GmbH and Charité - Universitaetsmedizin Berlin, Berlin, Germany; 3 Institute of Neuroradiology, University Luebeck, Luebeck, Germany; 4 Experimental and Clinical Research Center, Charité - Universitaetsmedizin Berlin and Max-Delbrück Center for Molecular Medicine Berlin, Berlin, Germany; 5 gfnmediber GmbH, Berlin, Germany; Universidade Federal do Rio de Janeiro, Brazil

## Abstract

Neuroaxonal degeneration in the central nervous system contributes substantially to the long term disability in multiple sclerosis (MS) patients. However, in vivo determination and monitoring of neurodegeneration remain difficult. As the widely used MRI-based approaches, including the brain parenchymal fraction (BPF) have some limitations, complementary in vivo measures for neurodegeneration are necessary. Optical coherence tomography (OCT) is a potent tool for the detection of MS-related retinal neurodegeneration. However, crucial aspects including the association between OCT- and MRI-based atrophy measures or the impact of MS-related parameters on OCT parameters are still unclear. In this large prospective cross-sectional study on 104 relapsing remitting multiple sclerosis (RRMS) patients we evaluated the associations of retinal nerve fiber layer thickness (RNFLT) and total macular volume (TMV) with BPF and addressed the impact of disease-determining parameters on RNFLT, TMV or BPF. BPF, normalized for subject head size, was estimated with SIENAX. Relations were analyzed primarily by Generalized Estimating Equation (GEE) models considering within-patient inter-eye relations. We found that both RNFLT (p = 0.019, GEE) and TMV (p = 0.004, GEE) associate with BPF. RNFLT was furthermore linked to the disease duration (p<0.001, GEE) but neither to disease severity nor patients' age. Contrarily, BPF was rather associated with severity (p<0.001, GEE) than disease duration and was confounded by age (p<0.001, GEE). TMV was not associated with any of these parameters. Thus, we conclude that in RRMS patients with relatively short disease duration and rather mild disability RNFLT and TMV reflect brain atrophy and are thus promising parameters to evaluate neurodegeneration in MS. Furthermore, our data suggest that RNFLT and BPF reflect different aspects of MS. Whereas BPF best reflects disease severity, RNFLT might be the better parameter for monitoring axonal damage longitudinally. Longitudinal studies are necessary for validation of data and to further clarify the relevance of TMV.

## Introduction

Increasing evidence documents that neuronal and axonal damage within the central nervous system (CNS) contributes substantially to the development of permanent disability in multiple sclerosis (MS) [1], [2], [3]. However, in vivo quantification and longitudinal monitoring of neurodegenerative processes remain a challenging task. Whole brain atrophy expressed by brain parenchymal fraction (BPF) is a frequently used MRI-based surrogate parameter for neurodegeneration within the CNS [4], [5], [6] and an inverse relation of BPF and disability progression has been consistently demonstrated [7], [8]. However, all MRI-based measures of brain atrophy have some important disadvantages. Besides limited availability, time consumption and costs, crucial confounders such as hydration status, inflammation, demyelination and age have to be accounted for [5]. Thus, a need for reliable, inexpensive and easily assessable complementary surrogate markers for neuroaxonal degeneration still remains. During the past two decades, optical coherence tomography (OCT) has emerged into a fascinating tool for the non invasive and reproducible in vivo studying of retinal neuroaxonal damage [9]. In MS patients, OCT has been consistently shown to detect thinning of the peripapillary retinal nerve fiber layer (RNFL) which is most probably due to a diffuse damage of retinal axons and at least in part independent of a previous optic neuritis (ON) [10], [11], [12]. Moreover, the determination of total macular volume (TMV) has been suggested as a marker for neuronal loss in MS patients [13]. Therefore, OCT might be a valuable tool for quantification and monitoring of both axonal and neuronal damage in MS [14], [15]. However, data on association between retinal nerve fiber layer thickness (RNFLT) and MS-determining parameters such as disease severity and disease duration are still inconsistent [15]. Whereas some studies found an association between RNFLT and disease duration [11], [16] both parameters were not related in other studies [17], [18]. Furthermore, data regarding the relation between OCT parameters and MRI measures for neurodegeneration are not yet consistent and a consensus on the most relevant parameter has yet to be reached [5], [15]. To date, the association between OCT parameters and BPF has been addressed in only two studies. Gordon-Lipkin et al. reported an association between RNFLT and BPF but not between TMV and BPF in a small cohort of 40 MS patients [19]. In another small study, Siger et al. found a correlation between RNFLT and BPF only in the subgroup without a history of ON [20]. Thus, the relation between OCT-based measures and BPF as an established MRI-based measure for neurodegeneration is not yet clear.

The aims of our prospective cross-sectional study were (i) to investigate the association between retinal neuroaxonal damage, measured by RNFLT and TMV, and cerebral neurodegeneration, measured by BPF, in a homogenous and sufficiently large cohort of patients with relapsing remitting (RR)MS and (ii) to evaluate the influence of important aspects such as age, disease duration, disease severity and ON history on RNFLT, TMV and BPF, respectively.

## Methods

### Ethics Statement

The study was approved by the local ethics committee of Charité Universitätsmedizin Berlin, Germany, and all participants gave informed written consent according to the 1964 Declaration of Helsinki.

### Patients

RRMS patients fulfilling the current panel criteria [21] were prospectively recruited from baseline visit of an ongoing clinical trial. All patients met the following criteria: age between 18 and 60 years, definite RRMS [21], expanded disability status scale (EDSS) between 0 and 6.5 [22], stable immunomodulatory treatment with glatiramer acetate for at least six months (this was an inclusion criterion of the ongoing clinical trial, patients were recruited from), no acute relapse (including optic neuritis) and no systemic steroid treatment within 30 days prior to enrolment. Medical history, particularly with respect to visual symptoms was taken from all study participants. All participants underwent a complete ophthalmologic examination including visual acuity testing, spheric and cylindric refractive error testing and non-contact tonometry. Patients with ophthalmologic disorders or medical conditions with impact on OCT parameters (e.g. diabetes, glaucoma) were not included.

### Optical Coherence Tomography

All OCT examinations were carried out on a Stratus 3000 OCT (OCT3, Carl Zeiss Meditec, Dublin, USA). RNFLT was measured using the “fast RNFL 3.4” protocol (software version 4.0). Three 3.4 mm diameter circular scans were acquired over 1.92 seconds. The OCT A-scan data were digitally exported in a blinded fashion and average RNFLT was calculated. As no specific real time volume scan protocol is available we used the “Fast Macular Thickness Map” protocol for determination of TMV which interpolates the area between the real time line scans to construct a circular model of the fovea and macula. Six radial line scans with 128 A-scans per line and a scan area of 6-mm diameter circle were acquired over 1.92 seconds. The maximum of 786.432 data points for fast protocols was obtained. For controlled manual export of the TMV data in mm^3^ we used the analysis protocol “Retinal Thickness/Volume Tabular”. A good quality image was defined as an image with generalized signal distribution, a reflectance signal from RNFL or retinal pigment epithelium strong enough to identify either layer, no missing parts caused by eye movements and a signal strength of ≥8 of 10 [23]. The segmentation line that defines the upper border of the retina was required to be on the internal limiting membrane and the lower border was required to be on the lower border of the RNFL (for RNFLT) or between the inner and outer photoreceptor layer of the RNFL (for TMV). Images not meeting these criteria were excluded.

### Magnetic Resonance Imaging

All MRI measurements were performed on a 1.5 Tesla scanner (Avanto, Siemens Medical Systems, Erlangen, Germany). A three-dimensional T1-weighted sequence (MPRAGE) was acquired from all participants according to the following protocol: TR 1.900 ms, TE 3.09 ms, TI 1,100 ms, flip angle 15°, resolution 1 mm^3^). Brain tissue volume, normalized for subject head size, was estimated with SIENAX [24], [25], part of FSL [26]. SIENAX starts by extracting brain and skull images from the single whole-head input data [27]. The brain image is then affine-registered to MNI152 space (using the skull image to determine the registration scaling) [28], [29]. This is primarily in order to obtain the volumetric scaling factor, to be used as normalization for head size. Next, tissue-type segmentation with partial volume estimation is carried out in order to calculate total volume of brain tissue [30].

### Statistical Analysis

The study was a prospective observational study with a primary endpoint defined by a regression approach with BPF as target and RNFLT and age as independent variables, allowing for the history of ON. The necessary sample size was calculated using assumptions from the study of Gordon-Lipkin [19]. For this calculation the reported, less pronounced R^2^ values from the whole MS group were used, since the RRMS subgroup's sample size was small and therefore of limited validity. Since no sample size calculation is available for that approach in Generalized Estimating Equations (GEE's) so far, the sample size calculation was based on linear multiple regression using the random model that supposes both target and predictor variables as random and should thus deliver a feasible estimation of the sample size required for GEE. A squared multiple correlation R^2^ of 0.21 for RNFLT plus age on BPF (Gordon-Lipkin *et al.*, 2007) would be detected with 95% power (alpha = 0.05, two-sided, k = 3 predictors) for n = 86 patients using the random model with an exact distribution. Considering a drop-out rate of 20% the final required sample size was estimated to n = 103 patients. Sample size was calculated using G*Power 3.1.2 (University of Duesseldorf, Germany) [31].

Normal distribution of outcome parameters BPF, RNFLT and TMV (the latter two considering an influence of history of ON) was tested using Shapiro-Wilk's test. For BPF, RNFLT and TMV the assumption of normality was not rejected.

The evaluation of the primary endpoint was accomplished by a GEE analysis with BPF as target variable and RNFLT and age as independent variables, taking into account the history of ON. Data of both eyes were included as repeated measures in order to account for inter-eye correlations. The working correlation matrix was defined as exchangeable (compound symmetry), i.e. the two eye-measurements were supposed equally correlated and independent from the sequence.

To address the diagnostic comparability of RNFLT or TMV with BPF in a second step, GEE analyses were performed with average RNFLT or TMV as independent variables and BPF as dependent variable while allowing for the history of ON, including again the data of both eyes as repeated measures and with an exchangeable corresponding working correlation matrix.

The influence of age, disease duration and disease severity (as expressed by EDSS) on BPF, RNFLT or TMV was analyzed in a third step: GEE analyses were performed with age, disease duration and EDSS (while allowing for the history of ON) as independent variables and with either BPF, RNFLT or TMV as dependent variable, using the same GEE-model specifications as before.

Since GEE in PASW 18 does not provide an output for standardized effect sizes or regression coefficients, we actualized this issue in the following way: age, disease duration, EDSS, RNFLT, TMV and BPF were transformed to standardized values and each GEE was performed with these z-values instead of the original values, thus achieving a better comparability with other data and understanding the effect sizes and associations.

To be able to compare our data to results reported by Gordon-Lipkin [19] the thinner RNFLT and TMV from each patient's eyes were selected (“minimum RNFLT” and “minimum TMV”) and used in partial correlations between BPF, minimum RNFLT and minimum TMV controlling for age. Since partial correlations do not account for inter-eye correlations, results from these tests should be interpreted carefully and used only in the context of comparability to the mentioned paper.

Significance in all tests was achieved with p<0.05. Beside the primary endpoint, all statistical evaluations should be understood as constituting exploratory data analysis, such that no adjustments for multiple testing have been made.

Statistical analysis was performed using PASW 18 (SPSS/IBM, Chicago, IL, USA).

## Results

### Cohort demographics

Our cohort included 104 patients (208 eyes) with RRMS. All patients underwent clinical evaluation, OCT examination and brain MRI. All patients with a complete data set were included in the subsequent analysis. 62 (60%) patients never had ON on either eye, whereas 29 patients (28%) had a history of unilateral and 13 patients (12%) of bilateral ON. Mean BPF, normalized for subject head size was 0.852 (SD 0.033), mean RNFLT was 95.2 µm (SD 14.2 µm) and average TMV was 6.769 mm^3^ (SD 0.489 mm^3^). Disease duration in this study was defined as time from establishment of MS diagnosis to enrolment in the trial. Patients' demographics and statistics are summarized in Table 1.

**Table 1:** Description of study cohort with demographic and disease parameters.

**RRMS-Patients**	n	104
**Eyes**	n	208
**Gender**	Male	35 (34%)
	Female	69 (66%)
**Age**	Mean (SD)	39.7 (8.8)
	Range	20–59
**Disease Duration [Months]**	Mean (SD)	68.2 (58.6)
	Range	3–269
**EDSS**	Median	2.0
	Range	0.0–6.0
**History of ON**	NON/NON	62 (60%)
	NON/ON	29 (28%)
	ON/ON	13 (12%)
**BPF**	Mean (SD)	0.852 (0.032)
	Range	0.77–0.922
**Average RNFLT [µm]**	Mean (SD)	95.2 (14.2)
	Range	46–133
**TMV [mm^3^]**	Mean (SD)	6.769 (0.489)
	Range	5.455–7.674

RRMS = relapsing remitting Multiple sclerosis; (N)ON = (non) optic neuritis; SD = standard deviation, BPF = brain parenchymal fraction, RNFLT = retinal nerve fiber layer thickness, TMV = total macular volume.

### Association of OCT parameters with brain atrophy

The primary endpoint of our study was defined as the association between BPF and RNFLT, using BPF as target and RNFLT and age as independent variables and taking into account the history of ON, which corresponds to the study by Gordon-Lipkin [19]. In our study, the association was evaluated by GEE analysis accounting for within-patient inter-eye dependencies. In our cohort, BPF was predicted by patients' age and RNFLT, however the standardized coefficient for the association between RNFLT and BPF was extremely low (table 2).

**Table 2:** Generalized Estimating Equations for the association of RNFLT with BPF as primary endpoint.

		Beta	stand. Beta	CI95% Low	CI95% High	p
**BPF**	**ON**					0.617
	**age**	−0.002	−0.457	−0.611	−0.303	<0.001
	**RNFLT**	<0.0001	<0.0001	<0.0001	<0.001	0.021

Results from GEEs with RNFLT and age as independent variables and controlling for history of optic neuritis and BPF as dependent variable. The standardized Beta was calculated as described in the methods section. RNFLT = retinal nerve fiber layer thickness, ON = history of optic neuritis, CI = confidence interval.

**Figure 1:**
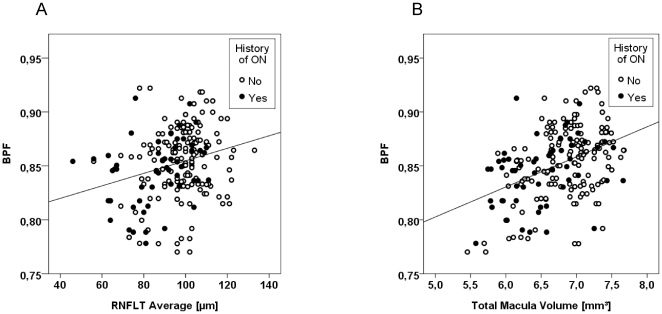
Association between BPF and OCT parameters in patients with RRMS. Patients (individual eyes) are labeled according to the history of optic neuritis (ON). Lines are derived from linear regression analyses with R^2^ given in parentheses. Statistical significance level was calculated by Generalized Estimating Equation models controlling for the history of ON. A) Retinal nerve fiber layer thickness (RNFLT) vs. BPF (0.073, p = 0.019). B) Total macular volume (TMV) vs. BPF (0.113, p = 0.001).

Next, we analyzed the associations between RNFLT or TMV and BPF by a GEE model, which only factors the history of ON and thus reflects better the diagnostic situation. GEE with BPF as target and RNFLT as independent variable and accounting for the history of ON showed a stronger though still weak association for RNFLT (table 3 and figure 1a). Interestingly, TMV was also associated with BPF when using the same model with TMV as independent variable (table 3 and figure 1b).

**Table 3:** Generalized Estimating Equations for the association of RNFLT or TMV with BPF.

	GEE controlled for ON			Partial Correlation controlled for age	
	Beta	stand. Beta	p	R	p
**RNFLT**	<0.0001	0.0001	0.019	0.269	0.006
**TMV**	<0.0001	0.0002	0.004	0.369	<0.001

Results from GEEs with RNFLT or TMV as independent values and controlling for history of optic neuritis and BPF as dependent variable are given in columns 2–4. The standardized Beta was calculated as described in the methods section. Additionally, partial correlation coefficients controlling for age are displayed in the last two columns to allow comparability to previous results [19]. P-values are given in parentheses. RNFLT = retinal nerve fiber layer thickness, TMV = total macular volume, ON = history of optic neuritis, GEE = Generalized Estimating Equations.

With respect to the comparability of our data to results reported by Gordon-Lipkin [19], we additionally performed partial correlation analyses controlling for age and using minimum RNFLT or minimum TMV and BPF as variables. In line with the GEE analyses, we found a significant but moderate correlation between RNFLT and BPF and between TMV and BPF (table 3).

### Influence of age, disease duration and severity

Having demonstrated that on the one hand both RNFLT and TMV associate with brain atrophy and on the other hand, in the same cohort age is strongly predictive for BPF, we asked whether the three parameters RNFLT, TMV and BPF are linked to distinct aspects of the disease such as age, disease duration and disease severity as determined by EDSS. Therefore, we performed GEE modeling with BPF, RNFLT or TMV as dependent variables and age, disease duration and EDSS as independent variables and correcting for history of ON. Data are presented in table 4. In summary, the analyses confirmed that BPF is substantially determined by both the patients' age (p<0.001) and EDSS (p<0.001). Notably, in our cohort BPF was not linked to disease duration. For RNFLT our data confirmed the impact of a previous ON as the history of ON was associated with a lower RNFLT (p<0.001). Importantly, the only other parameter that showed a significant impact on RNFLT was disease duration (p<0.001). Neither age nor EDSS were linked to RNFLT. For TMV the only determining parameter was ON history (p<0.001). TMV was neither linked to the duration or severity of the disease nor to the patients' age.

**Table 4:** Correlation between age, disease duration and severity with BPF, RNFLT and TMV.

		Beta	stand. Beta	CI95% Low	CI95% High	p
**BPF**	**age**	−0.001	−0.334	−0.499	−0.168	<0.001
	**duration**					0.098
	**EDSS**	−0.005	−0.223	−0.390	−0.057	<0.001
	**ON**					0.067
**RNFLT**	**age**					0.585
	**duration**	−0.073	−0.299	−0.471	−0.128	<0.001
	**EDSS**					0.201
	**ON**	−9.597	−0.675	−0.906	−0.444	<0.001
**TMV**	**age**					0.230
	**duration**					0.088
	**EDSS**					0.240
	**ON**	−0.300	−0.614	−0.818	−0.410	<0.001

Beta coefficients, standardized Beta coefficients, confidence interval for standardized beta coefficients and p values are provided as calculated by Generalized Estimating Equations accounting for inter-eye dependencies with age, duration, EDSS and history of optic neuritis as independent variables and RNFLT, TMV or BPF as dependent variables. For better clarity, coefficients are only given for factors that reached statistical significance. Standardized Beta was calculated as described in the Methods section. BPF = brain parenchymal fraction, RNFLT = retinal nerve fiber layer thickness, TMV = total macular volume, ON = history of optic neuritis, CI = confidence interval.

## Discussion

Investigating the associations between OCT parameters and BPF as an established MRI measure for neurodegeneration in a cross-sectional prospective study on 104 RRMS patients we here report an association between global brain atrophy and both thinning of the RNFL and reduction of macular volume. In line with previous studies, reduction of both average RNFLT and TMV was linked to the history of ON. Importantly, apart from the impact of ON, RNFL thinning correlated closely with disease duration whereas BPF was determined by age and disease severity. TMV associated neither with disease duration, disease severity nor age.

The strengths of our study are the large sample size, the prospective design, the homogeneity of our study cohort including exclusively RRMS patients on a stable immunomodulatory treatment, the evaluation of the influence of disease duration, disease severity and age on RNFLT, TMV or BPF in the same population, and a statistical approach taking within-patient inter-eye relations into account. On the other hand, with respect to our cohort characteristics which reflect patients with relatively short disease duration and mild to moderate disability, our results should not be uncritically transferred to MS populations with different characteristics.

Although OCT receives increasing attention as future tool for the detection and monitoring of neurodegenerative processes in MS the evaluation of the actual value of this technique remains difficult. This is mainly, because on the one hand, the interrelations between neurodegenerative processes in the retina and the brain are still under investigation and on the other hand, the impact of disease-related aspects such as duration and severity and disease-unrelated parameters such as age on OCT parameters is not yet clear [5], [15]. The only published two previous studies addressing the correlation of OCT parameters with BPF included only a limited number of patients (between 18 and 40) or inhomogeneous disease courses and did not account for within-patient inter-eye relations [19], [20]. In the present cohort we used a statistical model which allows adjusting for within-patient inter-eye relations and corrected for the history of ON, which we consider an appropriate approach in a population with different ON status. Our primary endpoint reflects the statistical model used by Gordon-Lipkin and was primarily defined for the estimation of the sample size. However, for the evaluation of associations between BPF and RNFLT or TMV we favor our second GEE model which does not account for disease duration, disease severity or age and thus represents a rather diagnostic than pathophysiological point of view. Since GEE does not provide standardized output for correlation coefficients we additionally calculated a “standardized Beta” in order to provide a better conception of the effect sizes. Using this model, the association between BPF and RNFLT was significant but weak (table 3, figure 1a). Interestingly, when applying the same statistical approach as Gordon-Lipkin et al., which however does not account for inter-eye dependencies, we also found an association between minimum RNFLT and BPF but with a moderate partial correlation coefficient (table 3), which is in line with Gordon-Lipkin [19]. In contrast to Siger et al. who found an association of RNFLT and BPF only in a cohort subgroup without ON [20], in our study the correlation was evident in the total cohort.

With respect to TMV our data (according to both GEE and partial correlation analyses; table 3) contrast Gordon-Lipkin et al., who did not find a correlation between TMV and BPF and suggested that “in a cohort of patients with MS with a mean duration of disease of approximately 10 years, TMV may be less informative than in a cohort with a longer history of MS” [19]. Our data rather indicate, that TMV in fact reflects global brain atrophy already after a mean disease duration of approximately five years and even in patients with a predominantly mild clinical disability (table 1) and suggest that damage to retinal ganglion cells occurs already in earlier phases of the disease. That in turn would be in line with grey matter damage detected early in the disease course by MRI or histopathology [32], [33], [34]. The differences in our data compared to previous data [19], [20] may be at least partially explained by the larger sample size in our study and the different cohort characteristics. The different effect sizes estimated by GEE and partial correlations might indicate, that at least in a cohort with a comparably short disease duration and mild disability, partial correlation analysis with minimum RNFLT/TMV not factoring inter-eye dependencies, might overestimate the strength of associations. It remains to be seen whether in a currently running longitudinal study factoring the changes within the parameters over time the standardized beta will conform to the partial r.

Numerous studies have addressed the relations between RNFLT, TMV or BPF with disease duration, disease severity, ON history and age in individual cohorts with different characteristics (summarized and meta-analyzed in two recent major review articles [5], [15]). However, data are still inconsistent. For example, a correlation between disease duration and thinning of RNFL has been reported in some studies [11], [16], [20], [35] whereas others did not find a correlation [17], [18]. Consequently, the ability of each marker RNFLT, TMV and BPF, respectively, to capture distinct aspects of the disease remains unclear. Evaluating the impact of disease duration, severity and age on each of the MRI and OCT parameters within the same cohort by GEE and correcting for the ON history, we here demonstrate that BPF might be a good parameter for the evaluation of the disease severity as, in line with previous reports [8], it associated best with the EDSS. On the other hand, our observations that in the same experimental setting RNFLT but not BPF were linked to the disease duration and that furthermore BPF but not RNFLT was substantially confounded by the patients' age, which is in line with previous reports [19], suggest that RNFLT is the better parameter for duration-related issues such as longitudinal monitoring in clinical trials. The relevance of TMV remains elusive, as TMV was not associated with any of the parameters. TMV captures not only the retinal nerve fiber layer, but also deeper layers of the retina, which in particular in combination with the use of time-domain OCT might render TMV a less specific parameter for neuroaxonal degeneration. Furthermore, as the sample size calculation was based on RNFLT this study was not powered to primarily investigate the role of TMV. Not surprisingly, our data moreover confirmed the impact of ON on RNFLT and TMV demonstrated in previous studies [12], [15].

In summary, our cross-sectional data on the association between both RNFLT and TMV with BPF point to a significant but weak association which was at least in our cohort independent of a previous ON. RNFLT and BPF but not TMV are linked to certain aspects of MS. Whereas BPF reflects in the first place the severity of the disease, RNFLT might be the better parameter for monitoring axonal damage longitudinally. Thus we conclude that in addition to BPF at least RNFLT is a promising complementary parameter to evaluate early neurodegenerative processes in RRMS patients. The eligibility of TMV as surrogate marker requires further evaluation. Longitudinal studies and studies on patients with a longer disease duration and higher disability are necessary to corroborate the relevance of these parameters and to clarify the remaining questions. We therefore suggest that both RNFLT and TMV should be included as standard secondary endpoints in clinical trials addressing neurodegeneration in MS.
